# Navigating a Path to Rifampicin Resistance in Tuberculosis

**DOI:** 10.1128/mbio.02952-22

**Published:** 2023-01-23

**Authors:** Sakshi Agarwal, Babak Javid

**Affiliations:** a Division of Experimental Medicine, University of California, San Francisco, California, USA

**Keywords:** antibiotic tolerance, differentially detectable, drug resistance, persister, rifampicin, tolerance, tuberculosis

## Abstract

For model bacteria, genetic drug resistance usually arises from antibiotic-tolerant subpopulations, but whether this is true for the globally important pathogen Mycobacterium tuberculosis—the cause of tuberculosis—is not known. Here, we discuss a recent article by Sebastian et al. (J. Sebastian, A. Thomas, C. Levine, R. Shrestha, et al., mBio 14:e0279522, 2023, 10.1128/mbio.02795-22) which leverages a robotic transwell microtiter experimental system coupled with deep sequencing of a barcoded library of M. tuberculosis to answer this question for rifampicin resistance. The authors investigate two distinct forms of antibiotic-tolerant subpopulations—classical tolerance, characterized by prolonged minimum duration of killing, and “differentially detectable” (DD) bacilli that are viable but can be recovered only in liquid medium as opposed to plating. They demonstrate that, indeed, resistance arises preferentially from both rifampicin-tolerant subpopulations, though earlier in the DD population. Use of barcoded libraries and parallel culture systems shows promise in investigating phenotypes mediated by minority subpopulations of bacteria such as development of antibiotic resistance.

## COMMENTARY

Tuberculosis (TB), caused by Mycobacterium tuberculosis is the world’s deadliest infectious disease, killing more than 1.6 million people annually. Drug-resistant tuberculosis (DR-TB), in particular rifampicin-resistant TB, causes a disproportionate number of deaths. But how does drug resistance arise? Work in other organisms, Escherichia coli and Staphylococcus aureus, suggested that drug resistance arose from subpopulations of antibiotic-tolerant bacteria (1, 2). Antibiotic tolerance describes a subpopulation(s) of genetically drug-susceptible bacteria that are not killed or are killed more slowly by concentrations of antibiotic sufficient to kill the bulk population (3).

Unlike for bona fide rifampicin resistance, which has only one cause—mutations in the drug’s cellular target, the β subunit of RNA polymerase—there are multiple forms of antibiotic tolerance, each due to distinct mechanisms. For example, in M. tuberculosis, rifampicin tolerance can be due to nonreplicating persistence, i.e., slow-growing and relatively metabolically inactive bacteria (4), errors in protein synthesis (5), macrophage infection (6), alterations in central carbon metabolism (7, 8), or exposure to rifampicin itself (9, 10). However, which of these, if any, give rise to rifampicin resistance in M. tuberculosis is unknown. In an article recently published in *mBio*, Sebastian et al. demonstrate that in M. tuberculosis, rifampicin resistance arises from distinct antibiotic-tolerant subpopulations (11).

One of the bottlenecks in linking tolerance to resistance, particularly in mycobacteria, which grow slowly and require special culture conditions, is that the traditional assay for antibiotic tolerance, time-kill curves (often referred to as minimum duration of killing [MDK]), even with some attempts at optimization (12), are still extremely time-consuming and laborious to perform. Furthermore, bulk methods such as MDK assays cannot easily identify from which distinct subpopulations eventual drug resistance arises. To address both of these limitations, Sebastian et al. developed a robotic transwell platform (transwell-tolerance-resistance [TTR]), coupled with a barcoded M. tuberculosis library (11). Unlike model organisms such as E. coli, in which time-kill curves are performed over a few hours, similar experiments with M. tuberculosis require drug exposure over many days and weeks. There is a concern that, over time, antibiotics may degrade and therefore the amount of active drug at later time points may not be the same as that at the start of the experiment. The transwell setup incorporated 24 upper wells, in which the M. tuberculosis was cultured, each paired with a lower well that allowed for exchange of fresh medium with or without antibiotics. Antibiotic concentrations were kept fairly constant via robotic medium exchange in the lower well at defined time points. The transwell design allowed rapid equilibration of antibiotic concentrations with the upper well, in which the bacteria were entirely confined. In this regard, the TTR system was similar to the hollow-fiber model (13). However, the TTR setup had one major advantage: incorporating a microtiter plate format allowed the researchers to examine the entire volume of each well at distinct time points, essentially representing multiple, parallel independent experiments.

Sebastian and colleagues investigated two forms of rifampicin tolerance. One was classic tolerance, as measured by prolonged time to kill compared with the bulk population; this subpopulation was measured by taking wells at defined time points, washing away antibiotic, and then directly plating onto antibiotic-free agar plates. Referred to as the “direct plating” (DP) subpopulation, it was used to assay those bacteria that were able to survive rifampicin (at 10 times the minimum concentration needed to kill the bulk population) and able to subsequently regrow on solid medium. They also measured a different bacterial subpopulation, referred to as “regrowth plating.” These “differentially detectable” (DD) bacteria are viable but cannot be recovered by conventional plating. They can nonetheless grow when inoculated into liquid medium. Previously, Saito et al. had demonstrated that exposure of nutrient-starved M. tuberculosis to rifampicin can result in DD M. tuberculosis that is antibiotic tolerant (9).

**FIG 1:**
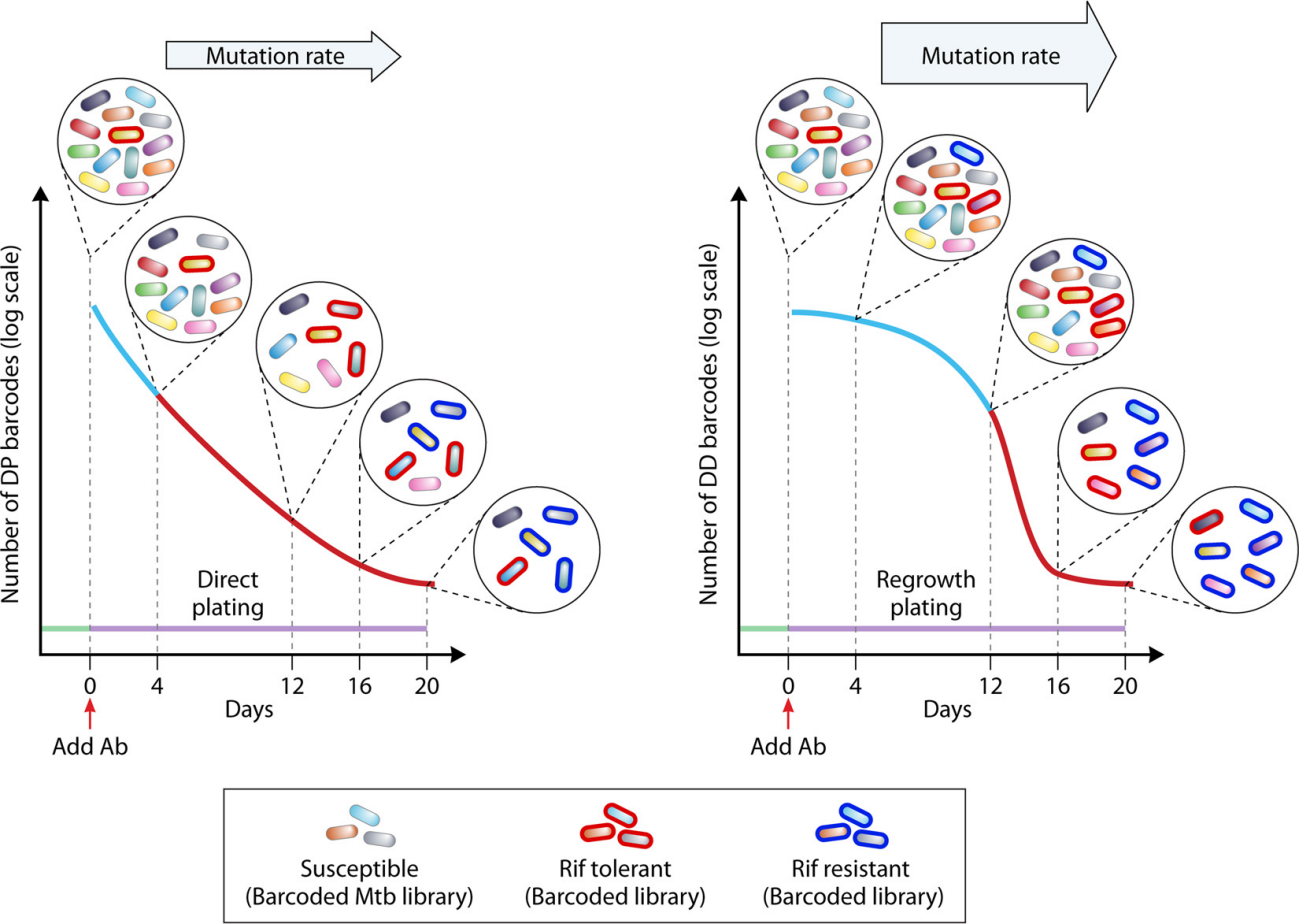
Drug-tolerant subpopulations facilitate the evolution of bona fide rifampicin resistance. Two distinct subpopulations of rifampicin tolerance were assayed from a barcoded library of M. tuberculosis using the TTR system. DP (direct plating) tolerant bacteria represent classical tolerance, i.e., bacteria that survive killing by the drug and are recovered by plating on antibiotic-free agar medium. Differentially detectable (DD) tolerant bacteria are recovered by regrowth initially in liquid medium, i.e., regrowth plating. Drug-tolerant (denoted by red border) subpopulations arise in both the DP (left) and DD (right) subpopulations but exhibit distinct dynamics. DP populations arise 12 days after initial antibiotic exposure, whereas DD bacteria exhibit an early-onset (day 4) but transient tolerance and then reemerge after day 16. Bona fide rifampicin resistance (denoted by blue border) emerged from both subpopulations but earlier in the DD population. Ab, antibiotic; Rif, rifampicin.

The authors performed their experiment twice with the same library of ~4,400 uniquely barcoded bacilli, with ~1,000 unique barcodes per experimental well. Sequencing the starting library and subsequently the library at defined points in the experiment allowed them to ask a number of questions: (i) does rifampicin resistance arise from rifampicin-tolerant subpopulations? (ii) is the tolerant subpopulation(s) preexisting? (iii) are the dynamics of tolerance and resistance similar between conventional tolerant and DD tolerant subpopulations? (iv) if rifampicin resistance arises from tolerant subpopulations, is it more likely to arise from one type of tolerant subpopulation than another (Fig. 1)?

As expected with bactericidal antibiotics such as rifampicin, there was a biphasic killing of M. tuberculosis upon drug exposure: initially a rapid 10-fold decline in population size over the first 12 days, followed by a slower decline from days 12 to 20, as measured by the DP population (Fig. 1). During the rapid-kill phase, sequencing of barcodes showed a relatively uniform decrease in barcode abundance, implying fairly uniform killing of the bulk population during this first period. However, subsequently, in the second, slow-kill phase (days 12 to 20), a small tolerant subpopulation was barely killed, while the bulk population continued to decline, albeit more slowly. Since drug exposure was initiated after dividing the seed library into individual wells, the authors could ask if tolerance arose before or after drug exposure. If the tolerant bacteria arose after drug exposure, one would expect that the bacteria that survived killing would be represented by distinct barcodes in each experimental well. Although this was true in the rapid-kill phase, the opposite was true in the persistent phase: there was a tendency toward shared “tolerant” barcodes between wells. Moreover, the “tolerant” barcodes were different between the two independent experiments. Together, these data suggest that certain bacteria are predisposed to tolerance, but this is not due to a genetic predisposition (since otherwise the barcodes would be the same between experiments); rather, the tolerance emerged as the seed culture was expanded prior to antibiotic exposure. Examining the DD population via regrowth plating, the authors identified a dynamic different than rifampicin tolerance: the initial rapid kill observed via DP was absent, but there was a more sudden decline between days 12 and 16, suggesting that DD bacteria had an earlier but transient tolerance to drug exposure and a subsequent, second tolerant phase from days 16 to 20 (Fig. 1).

Resistance arose earlier in DD subpopulations (by day 4) than in the DP population. There was also a strong association between tolerance-associated barcodes (both DP and DD) and subsequent development of bona fide rifampicin resistance. These “resistance-associated” barcodes were enriched >100-fold from the tolerant subpopulations compared with their representation in the starting cultures, strongly suggesting that resistance arises from preexisting tolerant subpopulations. Once again, comparing resistance-associated barcodes between the two experiments revealed completely distinct barcodes, i.e., the propensity for resistance arose subsequent to the aliquoting of the library into seed cultures.

Taken together, the TTR system allowed Sebastian et al. to address the critical questions outlined above: tolerance to rifampicin appears to arise prior to drug exposure. Assaying classical tolerance and DD tolerance revealed very distinct kill dynamics between the two subpopulations—remarkably, DD bacteria demonstrated a strong but transient early tolerance to rifampicin killing. Resistance arose from both DP and DD subpopulations, but earlier in the latter. However, a number of questions still remain. Given that TB is treated with several antibiotics simultaneously, are the tolerant subpopulations that arose from single-drug therapy equally likely to arise under multidrug therapy? Furthermore, for pragmatic purposes, the authors chose to study an auxotrophic strain of M. tuberculosis (mc^2^6230) that could be cultured under biosafety level 2 conditions (14). Given the demonstrated critical role of central carbon metabolism to multiple mechanisms of rifampicin tolerance in M. tuberculosis, it is not certain if the experiment fully captures the dynamics of tolerance in wild-type M. tuberculosis. Finally, it is possible that both different strains of clinical isolates of M. tuberculosis and immune pressure from the host (15) can contribute to diverse mechanisms of antibiotic tolerance. It remains to be seen if these additional drivers of diversity further complicate the convoluted path M. tuberculosis takes from drug susceptibility to tolerance to resistance.
